# Fake news on Facebook and their impact on supply chain disruption during COVID-19

**DOI:** 10.1007/s10479-022-05124-1

**Published:** 2022-12-19

**Authors:** Mohammad Alamgir Hossain, Md. Maruf Hossan Chowdhury, Ilias O. Pappas, Bhimaraya Metri, Laurie Hughes, Yogesh K. Dwivedi

**Affiliations:** 1 School of Accounting, Information Systems, and Supply Chain, RMIT University, Melbourne, VIC 3000 Australia; 2 RMIT Business and Human Rights (BHRIGHT) Centre, RMIT University, Melbourne, VIC 3000 Australia; 3 UTS Business School, University of Technology Sydney, Broadway, NSW 2007 Australia; 4 University of Agder, Kristiansand, Norway; 5 Norwegian University of Science and Technology, Trondheim, Norway; 6 Indian Institute of Management Nagpur, Dahegaon, India; 7 Digital Futures for Sustainable Business & Society Group, School of Management, Swansea University, Bay Campus, Swansea, UK; 8 Digital Futures for Sustainable Business & Society Group, School of Management, Swansea University, Bay Campus, Fabian Bay, Swansea, SA1 8EN Wales UK; 9 Department of Management, Symbiosis Institute of Business Management, Pune & Symbiosis International (Deemed University), Pune, Maharashtra India

**Keywords:** Fake news, Social media, COVID-19, Supply chain disruption, Resilience, Panic buying

## Abstract

Social media (SM) fake news has become a serious concern especially during COVID-19. In this study, we develop a research model to investigate to what extent SM fake news contributes to supply chain disruption (SCD), and what are the different SM affordances that contribute to SM fake news. To test the derived hypotheses with survey data, we have applied partial least square based structural equation modelling (PLS-SEM) technique. Further, to identify how different configurations of SC resilience (SCR) capabilities reduce SCD, we have used fuzzy set qualitative comparative analysis (fsQCA). The results show that SM affordances lead to fake news, which increases consumer panic buying (CPB); CPB in turn increases SCD. In addition, SM fake news directly increases SCD. The moderation test suggests that, SCR capability, as a higher-order construct, decreases the effect of CPB on SCD; however, neither of the capabilities individually moderates. Complimentarily, the fsQCA results suggest that no single capability but their three specific configurations reduce SCD. This work offers a new theoretical perspective to study SCD through SM fake news. Our research advances the knowledge of SCR from a configurational lens by adopting an equifinal means towards mitigating disruption. This research will also assist the operations and SC managers to strategize and understand which combination of resilience capabilities is the most effective in tackling disruptions during a crisis e.g., COVID-19. In addition, by identifying the relative role of different SM affordances, this study provides pragmatic insights into SM affordance measures that combat fake news on SM.

## Introduction

Fake news is a ubiquitous term, encompassing misinformation, disinformation, and mal-information (Carson, 2021) and can be defined as the false or fabricated or camouflaged information or rumour, either deliberate or accidental, that misleads people (Muigai, 2019). The frequency and severity of fake news is a global problem (Robinson et al., 2019). The most deleterious fake news circulation has been observed during COVID-19 pandemic (hereafter ‘COVID-19’) (Pennycook et al., 2020). It has not only resulted public fear and confusion but also hundreds of deaths (Coleman, 2020; Islam et al., 2020; Naeem et al., 2021). Online social media (SM) platforms became an easy and effective mechanism for the creation and spread of fake news (Islam et al., 2020; Wisker, 2020). During COVID-19, the speed of the spread of fake news was quicker than that of the virus itself (thus named “infodemic”) and became a serious concern (Bermes, 2021; Park, Fisher, Lee, et al., 2021a). For instance, “India’s battle against the coronavirus has many obstacles—large crowds, a stretched health system and inadequate infrastructure … But beyond these, familiar foes are rearing their heads: misinformation and fake news …. through its vast social media networks.” (Purohit, 2020).

SM fake news has severe adverse effects including misconstrued understandings of the situation and downplay factual information, creating and propagating speculations and chaos, and eventually affecting the effectiveness of containment strategies (Coleman, 2020; Nyilasy, n.d.; van Der Linden et al., 2020). Specifically, SM fake news during COVID-19 (e.g., about crises of essential products) has created psychological stress, intensified behavioural responses of consumers e.g., panic buying (Arafat et al., 2021; Bermes, 2021; Loxton et al., 2020). Studies suggest that COVID-19 pandemic itself (e.g., Bag et al., 2021; Dubey et al., 2021) as well as SM fake news (Barua et al., 2020; Butt, 2021; Islam et al., 2020; Zheng et al., 2021) have disrupted supply chains globally, mostly through a sudden surge in demand ‘by surprise’ (Ho, 2020). Given its adverse consequences, the impact of SM fake news on SC disruption (hereafter ‘SCD’) has become an important and emerging research topic (Dulam et al., 2021). Understanding the threat COVID-19 SM fake news poses to SC could be important for various stakeholders such as suppliers, distributors, retailers, consumers and regulators. In a different note, SM fake news is possible because of SM affordances (Apuke & Omar, 2021). Therefore, understanding this current phenomenon from an affordance perspective is vital in order to shed light onto the measures that effectively reduce fake news by designing effective pragmatic interventions. In other words, looking into the causes of the SM fake news along with its impact may provide us a more complete picture of the phenomenon.

Against the backdrop, this current study investigates how SM fake news cause and/or heighten SCD during COVID-19. Drawing on *moral panic theory* (Cohen, 2011), we propose that the fake news on SM platforms develop irrational and widespread panic e.g., CPB during COVID-19, which ultimately leads to SCD. Moreover, we use the *affordance theory* (Chemero, 2003; Cohen, 1972) to delineate how SM (namely Facebook) affordances provide a favourable condition for and thus contribute to SM fake news. Specifically, we endeavour to answer two primary research questions:

- **Research question 1**: To what extent social media fake news contributes to perceived supply chain disruption during COVID-19?
- **Research question 2**: What are the social media affordances that contribute to social media fake news?

This study responds to several calls for research. Specifically, Dulam et al. (2021) criticise that, even though change in consumer behaviour (e.g., panic or over buying) in times of disaster is a major cause of SCD, the current studies have ignored it; therefore, more research is required. Similarly, Zheng et al. (2021) suggest more research to explore consumer behaviour during SCD. Hence, we look on the impact of fake news on SCD during COVID-19, which contributes to theory and practice in three distinct ways. *First*, this work advances our knowledge on to what extent SM fake news produce SCD through CPB. To reduce the effect of CPB on SCD, we suggest different configurations of supply chain resilience (SCR) capabilities of firms (e.g., high visibility and collaboration). *Second*, this work enriches our understanding by identifying what SM affordances contribute to fake news. We test four SM affordances (i.e., information-sharing, connectivity, editability, and association-denial) that influence users’ evaluation of SM platform as a place for entertaining fake news. Although prior research has related the SM affordances with users’ behaviour (e.g., Cabiddu et al., 2014; Chan et al., 2019; Treem & Leonardi, 2013), this current study identifies the SM affordances that contribute to fake news. *Finally*, from theoretical perspective, our study extends existing knowledge by integrating *moral panic theory* and *affordance theory* in a single nomological model to understand the antecedents and outcomes of SM fake news during COVID-19. It also offers pragmatic implications to SC professionals and SM administrators.

The rest of the paper is organised as follows. The next section discusses the underpinning theories and literature upon which the research model of the current paper is developed followed by presenting the research model and the hypotheses. This is followed by a discussion of our research methodology, followed by presenting the data analysis techniques and results. We then offer a discussion of the results and implications for research and practice. The paper concludes with a conclusion.

## Background literature

SCD during COVID-19 has been driven mostly by social panic among the consumers where SM has been used as the ‘instruments of panic production’ (Walsh, 2020). Considering the theoretical implications of *moral panic theory*, it is a viable theoretical perspective for explaining to what extent SM fake news contributes to consumer panic. “Moral panic theory has been extensively applied in media studies … to analyse the role of media in highlighting social problems” (Makoza & Chigona, 2016, p. 2) during moral panic instances (Cohen, 2011). Using moral panic theory, studies examine how media (e.g., website, blogs, forums and other social media) contribute to, for instance, anti-immigrant sentiments (Flores-Yeffal et al., 2011) and anti-asylum-seekers movement (Martin, 2015), and to social panic during COVID-19 (e.g., DeVore et al., 2021). In a different note, studies (e.g., Apuke & Omar, 2021) explain how SM affordances (e.g., (mis)information sharing) are perceived to contribute to fake news circulation resulting moral panic. Accordingly, this section discusses SCD, followed by moral panic theory, and ends with a discussion on SM affordance.

### Supply chain disruption (SCD)

SCD refers to uncertain or unintended but consequential situation or event that interrupts SC operations e.g., inbound flow of goods from suppliers, production and distribution of finished goods to consumers on right time, quality and quantity (Bode et al., 2011; Craighead et al., 2007). Glaringly, more than one out of two companies experience SCD each year (Business-Continuity-Report, 2019) and consequent both short and long-term impacts. Therefore, both firm managers as well as researchers should take SCD seriously.

Understanding the sources of disruption and their nature is salient in setting mitigation strategies (DuHadway et al., 2019). SC literature suggest that SCD may originate from supply side disruptions (Zsidisin & Wagner, 2010), catastrophic disruptions (Jia et al., 2020), demand side disruptions (Chen & Xiao, 2009), production disruptions (Ivanov et al. 2017), and infrastructure disruptions (Chopra & Sodhi, 2014). Other studies (e.g., Shekarian & Mellat Parast, 2021) report that SCD are either external or internal to firms. The external disruptions are less frequent, less controllable but with severe impact and include economic downturns, technology changes, labour strikes, natural calamities, and terrorist attacks (Shekarian & Mellat Parast, 2021). Alternatively, internal disruptions (i.e., operational) are more frequent, controllable, and predictable which may arise from supply–demand coordination problems (Shekarian & Mellat Parast, 2021). The SCD during COVID-19 combines both. For example, retail supply chain managers have faced huge challenges relating to demand supply coordination during COVID-19 due to severe demand oscillation of necessary goods. On the other hand, external factors such as transportation shortage and lockdown have substantially affected the movement of goods during the pandemic (Burgos & Ivanov, 2021; Dulam et al., 2021).

During COVID-19, supply–demand disturbances at the global and local business arena have collapsed a large number of SC; in many instances, SC managers struggled to manage such disruptions (Ivanov, 2020). Specifically, during COVID-19, consumers were hoarding and over-purchasing essential goods, which created huge supply shock in various SC. Further, border and movement restrictions leading to labour shortages, reduced production capacity, shortage of raw materials, and transportation have intensified SCD (Hobbs, 2021; Paul et al., 2021). This has further been intensified with the SM fake news. Accordingly, research on understanding the cause of SCD and designing their mitigation tactics are suggested (Ivanov, 2020).

### Moral panic theory and the current context

“‘Moral panic’ is a sociological concept that seeks to explain a particular type of overreaction to a perceived social problem” (Rohloff & Wright, 2010, p. 404), “exaggerated and amplified by the media” (Kent, 2007). According to Cohen (1972), the architect of the *moral panic theory,* the term *moral panic* represents a situation when media creates a ‘folk devil’, “and the public demand of the authorities that something is done about it” (Frothingham, 2021). Media is an essential component of this theory, if not the main, where it behaves as the ‘facilitator’ of division, hostility and panic (Walsh, 2020). Most of the times, the media presents news in such a way so that the social norms are challenged, and people engage in acts out of panic, which are mostly irrational.

*Moral panic* is a mass movement, based on false or exaggerated news, against a person (or object), which is more than that person (or the object) deserves or the actual threat it offers (Rohloff & Wright, 2010). It is a widespread fear, often irrational, of threat to society's morality, interests, and safety, defined by the media (Cohen, 1972). In other words, a moral panic is an exaggerated reaction of public concern, implanted by media, over the values and safety of the society (Frothingham, 2021). In short, moral panic is the public concern (to the minimum) and mass hysteria (to the extreme) created by media against an object where the mass public believe whatever is being reported on is occurring everywhere in society (Rohloff & Wright, 2010).

*Moral panic theory* consists five sequential stages (Crossman, 2019), which we explain with the context of this study. First, someone or something e.g., the unprecedented COVID-19 is defined (or farmed) as a threat to interests or safety of a given society. It is a situation where public fears greatly exceed the actual threat COVID-19 offers (Frothingham, 2021). Second, this threat is depicted in an easily recognisable and simplistic form by the media. Studies inform that the spread of fake news, misinformation, myths and rumours via SM during COVID-19 have increased (Ahmad & Murad, 2020; Al-Zaman, 2021; Arafat et al., 2021; Park, Fisher, Lee, et al., 2021a). Third, a rapid and extensive public concern is developed by the way the media portrays the threat. During COVID-19 pandemic, mandatory lockdowns and its associated rumours on SM platforms have created mass anxiety (Al-Zaman, 2021), fear and panic worldwide (Ahmad & Murad, 2020; Arafat et al., 2021). This resulted panic buying of toiletries, dry food etc. Fourth, in response to such chaos, the relevant authorities and policymakers develop new laws or policies. To control panic behaviours, government agencies have adopted measures ranging from legislative action and raised public awareness to fight with fake news (Robinson et al., 2019). Similarly, supermarket officials made regular announcements and installed policies e.g., rationing, non-return of sold goods. Fifth and finally, the panic recedes or results in social changes. Countries and consumers now understand that they need to deal with the coronavirus and thus are relatively less participative to COVID-19 panic.

As moral panic is short-termed and present-centred (Rohloff & Wright, 2010), it is suitable to explain the current phenomenon given that public fear and panic behaviour resulting from COVID-19 fake news do not last long, however, may repeat. Using this theory, DeVore et al. (2021) have shown that, during COVID-19, one-third of media coverage in China, South Korea, and the United States have contributed to moral panic. They further suggest, “The moral panic framing tended to stress … the lack of facilities to adequately respond to the virus as well as negative impacts on the economy” (p. 33). Walsh (2020) summarises as, “the role of digital communication in spreading fake news and inciting panic was on full display in initial reactions to the novel coronavirus (COVID-19)” (p. 847).

### Affordance theory and social media

Affordance theory posits that “behaviour is regulated with respect to the affordances” (Chemero, 2003, p. 183). Affordance refers to ‘opportunities for action’ (Gibson, 1977). It suggests the convergence of the properties of an object or environment that permit certain behavioural action (Cabiddu et al., 2014) and “the possible presence of” the actor “that can actualise them [the features]” (Chemero, 2003, p. 183). “It belongs neither to the technology nor to the user but to the interrelation between the purposes of the user and the capabilities of the technology” (Sun et al., 2020, p. 2). Specifically, ‘‘the concept of technology affordance refers to an action potential, that is, to what an individual or organization with a particular purpose can do with a technology or information system’’ (Majchrzak & Markus, 2012, p. 1). *Affordance* is hence the artefact that a technology (e.g., SM) allows people to perform some actions, which is “almost synonymously with the features of technology” (Bucher & Helmond, 2017, p. 11). In summary, affordances is “the mutuality of actor intentions and technology capabilities that provide the potential for a particular action” (Majchrzak et al., 2013, p. 39).

*Affordance theory* suggests that, the latent capabilities of a technology can be transformed into manifest potentiality only when are leveraged within a specific set of actions (Majchrzak et al., 2013). Hence, “rather than being exclusive properties of people or the technology” (Pee, 2018, p. 27), *affordance* suggests the symbiotic relationship between the capability of the technology and the actor action (Majchrzak et al., 2013). Conventional media are relatively more centralized, formal, permanent, and controlled as compared to online SM that allow more power to the users. In other words, SM can afford user behaviours that have been difficult or impossible to achieve with traditional media, which sometimes result in deviant behaviour of the users including bullying others (Chan et al., 2019) and creating and disseminating fake news (Apuke & Omar, 2021). For instance, the *editability* affordance of some SM (e.g., Facebook, Instragram) permits users to modify a content progressively (i.e., after it is posted), which is not possible in conventional media.

## Research model and hypotheses

### Fake news on social media, consumer panic buying, and supply chain disruption

According to the moral panic theory, while the issue in question may be real but the seriousness and extent of it is exaggerated, which may create social panic. In general, during heightened uncertainties, people may behave differently than usual. But, the widespread exaggeration of fake news during uncertainties may cause consumers to overreact and over-purchase i.e., panic buying (Loxton et al., 2020; Pennycook et al., 2020). Consumer panic buying (CPB) is a social psychological phenomenon, which has long been recognised (Sterman & Dogan, 2015) in several crises since twentieth century (Bentall et al., 2021). It is “an indiscriminate consumer behaviour” (Loxton et al., 2020, p. 168) which causes over reacting and over purchasing of essential goods due to the presumption of supply shock (O'Connell et al., 2021; Pennycook et al., 2020). SM fake news-led panic buying has drawn much attention to the literature in the wake of COVID-19. Recent studies (e.g., Ahmad & Murad, 2020; Ghaffary & Heilweil, 2020; Naeem & Ozuem, 2021; O'Connell et al., 2021) find that SM fake news heighten panic buying of essential goods during COVID-19. Thus, our first hypothesis, surrounding the alteration to typical patterns of consumers’ buying behaviour because of SM fake news during COVID-19, is:

- **H1a.** Perceived fake news on social media about COVID-19 increases consumer panic buying.

We further hypothesize that, not all fake news but the ones that people believe as genuine would lead to CPB. Here, *believability* is the degree of believing, which indicates the likelihood of users to believe and act on fake news when they encounter such news (Kumar et al., 2021). Kim and Dennis (2019, p. 1029) suggest that “believability … [of news] can affect the action of users”. In other words, user behaviour is influenced by the believability of the news. In the current context, based on believability, users can either ignore the news or act on it e.g., CPB. Therefore, we postulate that, the relationship between fake news and CPB is not static but contingent upon the believability of the news (Kim & Dennis, 2019). Therefore:

- **H1b.** The strength of the relationship between perceived fake news and consumer panic buying is moderated with the perceived believability of such news.

CPB causes “supply shortfalls and supply chain difficulties” (Loxton et al., 2020, p. 168). The state-wide lockdowns (and sometimes scam around these) during COVID-19 have triggered CPB at different outlets e.g., supermarkets, which increased SCD (Ahinkorah et al., 2020). Sim et al. (2020) reiterates that, CPB of a wide range of household commodities during COVID-19 has led to SCD in many countries. Dulam et al. (2021) too found that CPB has been a major cause for the severe shortage of products in retail stores in almost every sector. They further explain, “panic buying of a product leads to a sudden increase in demand, which creates mayhem along the SCs of the retail industries. This disruption progresses to initiate further panic buying, which converts into a vicious circle” (Dulam et al., 2021, p. 4371). In short, recent studies suggest that CPB creates SCD (e.g., Dulam et al., 2021; Zheng et al., 2021). Thus:

- **H2.** Consumers panic buying increases perceived supply chain disruption.

### Supply chain resilience capabilities

A resilient SC ensures smooth supply of products to the consumers (Dulam et al., 2021). Given the potential impacts of SCD, the need for designing resilient SC is of paramount importance (El Baz & Ruel, 2021; Kapoor et al., 2021; Katsaliaki et al., 2021). In SC literature, resilience has been explained either as capability or strategies to mitigate SCD. Extant studies (e.g., Chowdhury & Quaddus, 2016, 2017; Ponomarov & Holcomb, 2009) define SCR as the capability to manage disruptions. This is supported by Blackhurst et al. (2005), who have identified resilience as a capability to anticipate, adapt and promptly respond to unpredictable events. Conversely, other studies (e.g., Chowdhury & Quaddus, 2015; Kapoor et al., 2021) denote SCR as strategies to mitigate SCD. We adopt the former approach.

In combating SCD, SC studies guide a shift away from traditional risk management thinking (reactive tactic) towards building SCR capabilities (proactive thinking) (Katsaliaki et al., 2021). Especially during COVID-19, it has been evident that an on-time intervention with right resilience capabilities is essential for SC partners in tackling the negative consequences of SCD (Prentice et al., 2020; Zheng et al., 2021). Explaining SCR, extant scholarship comprehends various capabilities including flexibility, visibility, redundant/buffer capacity, collaboration, adaptability, efficiency, anticipation, and recovery (Chowdhury & Quaddus, 2016, 2017; Jüttner & Maklan, 2011; Pettit et al., 2013). In addition, different organisational capabilities including risk management capabilities (Bag et al., 2021), big-data analytics capability (Yu et al., 2021), and alliance management capabilities (Dubey et al., 2021) can increase SC resilience. In congruence with Chowdhury and Quaddus (2016) and Jüttner and Maklan (2011), we propose four capabilities in explaining SCR including flexibility, visibility, collaboration, and developing redundant/buffer capacity.

*SC flexibility* is considered as an essential resilience capability for firms to mitigate disruptions emanating from uncertain events (Pettit et al., 2013) because a flexible SC can adjust its operations based on the situational demand (Kapoor et al., 2021; Rojo et al., 2018). The role of SC flexibility has also been evident in minimizing the COVID-19 inflicted disruptions (Hobbs, 2021; Kovács & Falagara Sigala, 2021). Zsidisin and Wagner (2010) posited that SC flexibility moderates the impact of supply-side disruptions and the sources of disruptions. Drawing on the review of aforementioned literature, we postulate that SC flexibility minimizes the impact of panic buying on SCD. Therefore, we suggest:

- **H3a.** The greater the supply chain flexibility, the less the impact of consumer panic buying on perceived supply chain disruption.

*Visibility* in SC affirms that all parties in SC share their operational information so that each member in the chain gets access to required information on right time for decision-making (Messina et al., 2020). *SC visibility* is salient for SCR to mitigate disruptions (Blackhurst et al., 2011; Chowdhury & Quaddus, 2016; Jüttner & Maklan, 2011). Hamadneh et al. (2021) report that due to the lack of visibility of stocks, downstream SC members may start panic buying during crises. In this regard, Butt (2021) suggests that SC partners should work on visibility to minimize SCD, specifically during COVID-19. Based on the above studies, we argue that SC visibility helps reducing the severity of impact of CPB on SCD. Therefore, we suggest:

- **H3b.** The greater the supply chain visibility, the less the impact of consumer panic buying on perceived supply chain disruption.

*SC collaboration* enhances synergistic relationship among SC members, facilitates joint decision making and enables information sharing which are effective in the readiness, response and recovery process against disruptions (Scholten & Schilder, 2015; Whipple & Russell, 2007). Several studies in SC management literature documented that SC collaboration is effective in mitigating SCD (e.g., Chowdhury & Quaddus, 2017; Pettit et al., 2013; Shekarian & Mellat Parast, 2021). Similarly, recent studies (e.g., Kapoor et al., 2021; Sharma et al., 2021) highlighted that collaboration efficiency is pivotal for enhancing resilience and managing retail SCD. Likewise, Friday et al. (2021) suggest that SC collaboration, to manage optimal inventory and build a resilient SC, is potent to attenuate stock-out risks during COVID-19. Hence, we hypothesize that:

- **H3c.** The greater the supply chain collaboration, the less the impact of consumer panic buying on perceived supply chain disruption.

*Redundant capacity* refers to keeping back up capacity or resources so that organizations and their SC have resistance against disruptive events to minimize their impact (Katsaliaki et al., 2021). Redundant capacity minimizes expected sales loss, improves service level while firms that do not apply redundant capacity are susceptible to disruptions (Kamalahmadi et al., 2021). Numerous evidence show that redundant capacity help minimizing the impact of SCD (e.g., Chowdhury & Quaddus, 2017; Kamalahmadi et al., 2021; Pettit et al., 2013). Tomlin (2006) finds that when the disruption is frequent but short, a safety inventory/stock is highly effective for mitigating disruptions. Similarly, Alikhani et al. (2021) have found that redundant capacity is effective in enhancing SCR and mitigating SCD amid COVID-19-led disruptions. Aligning with prior studies, we assert that redundant capacity is salient for SCR to minimize the impact of SCD due to CPB. Hence, we posit that:

- **H3d.** The greater the redundant capability, the less the impact of consumer panic buying on perceived supply chain disruption.

### Social media affordances enabling fake news on social media

SM affordances are crucial for their use and misuse (Bucher & Helmond, 2017); therefore, studies describe SM’s role from an affordance perspective, which is advantageous for theory. For instance, Majchrzak et al. (2013) conceptualise four SM affordances representing different ways to engage publicly-visible knowledge conversations: meta-voicing, triggered attending, network-informed associating, and generative role-taking affordance. Similarly, Cabiddu et al. (2014) identify that SM provide three affordances for tourism service providers including persistent, customized, and triggered engagement with customers. From extant studies, Treem and Leonardi (2013) suggest four ‘relatively consistent affordances’ of social media: visibility, persistence, editability, and association. This is further extended by Chan et al. (2019) who suggest four SM affordances that include accessibility, information retrieval, editability, and association. Based on the prior studies on SM affordances (Apuke & Omar, 2021; Chan et al., 2019; Treem & Leonardi, 2013), we posit that four SM affordances^1^—information-sharing, connectivity, editability, and association-denial affordance—stimulate fake news on SM.

*Information-sharing affordance* can be defined as the extent to which SM users believe that SM offer the opportunity to share information with other users on the platform (Apuke & Omar, 2021). SM provide the opportunity to their users to create a post or (fake) news with least effort (Ahmad & Murad, 2020), and the news spreads when others share it (Kim & Dennis, 2019). “With the affordance provided by social media, sharing news or information has become easier as people can take part in the creation and dissemination of information” (Apuke & Omar, 2021, p. 4). While the information-sharing affordance permits users to share useful information (Wardle, 2020), unfortunately, the fake news have flourished through SM during COVID-19 (Laato et al., 2020). Recently, Apuke and Omar (2021) find that, SM’s information-sharing affordance increases SM fake news on COVID-19; therefore, we propose:

- **H4a.** Information sharing affordance is positively associated with fake news on social media.

*Connectivity affordance* can be defined as the extent to which SM users believe that SM offer the opportunity to connect with other users on the platform. Connectivity is a key defining affordance of SM (Pee, 2018), which enables SM users to expand their network (Weber & Haseki, 2021). SM e.g., Facebook has the capability of recommending friends and potentially relevant connections based on a user’s profile or activity (Pee, 2018). This affordance enables users to have a greater reach, expand their network and communicate contents. It has been evidenced that during COVID-19 lockdowns and restrictions, the use of SM has increased mostly to access news and social connectivity (Park, Fisher, Lee, et al., 2021a). As soon as some interesting news on COVID-19 comes, some users tend to connect with others in their network and beyond by sharing it (Apuke & Omar, 2021). In the absence of such easy connectivity, they probably would not so e.g., by sending emails or calling friends about the news. Therefore, we postulate that:

- **H4b.** Connectivity affordance is positively associated with fake news on social media.

*Editability*, a core affordance of some SM (Weber & Haseki, 2021), is defined as the extent to which users believe that SM offer the opportunity to manipulate the content that they posted, commented on, and/or shared on the platform (Chan et al., 2019; Pee, 2018). This affordance makes it possible for users to edit or revise own content. “For instance, Facebook allows users to edit descriptions of their posts or even delete contents published on their walls” (Chan et al., 2019, p. 586). This affordance, thus, allows Facebook users to deny their acts of posting and/or sharing misinformation by manipulating (e.g., erasing, editing). A survey in USA shows that 68% SM users delete their prior posts that do not represent their ‘thoughts’ anymore while 43% to protect their personal reputation (Statista, 2021). Similarly, to avoid embarrassment, SM users edit or delete their posted posts with information on COVID-19, which later they found false or misleading (Wong, 2021). For platform guardians, this affordance makes tracing fake news difficult; consequently, fake news increases. Therefore, we hypothesize that:

- **H4c.** Editability affordance is positively associated with fake news on social media.

In SM context, *association affordance* refers to the possibility of connections between individual users and their content (Pee, 2018; Treem & Leonardi, 2013). Lately, Chan et al. (2019) define it as “the extent to which a user believes that an SNS [social networking site] offers the opportunity to share responsibility for his or her post with other users who interact with the post on the platform” (Chan et al., 2019, p. 586). This latter definition actually refers to the denial of association and associated accountability; we call it *association-denial affordance*. This affordance allows SM users the denial of accountability for being associated with a fake content and sole responsibility for carrying out the action by shifting responsibility to others who interacted (e.g., shared, liked or commented). Further, others may shift the responsibility to others saying that they have not created the content but merely shared. The association-denial affordance complicates to make a user accountable for creating/propagating fake news; consequently, fake news increases. Therefore, we hypothesize that:

- **H4d.** Association-denial affordance is positively associated with fake news on social media.

**Fig. 1:**
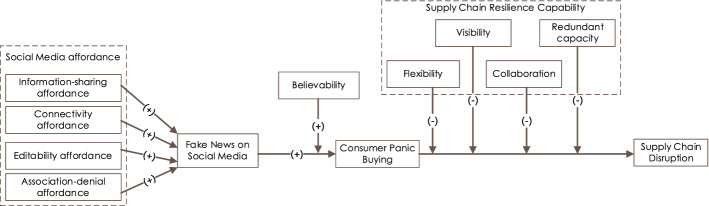
The research model

The research model is presented in Fig. 1.

## Research methodology

### Measures

This study has applied an anonymous, self-reported online survey-based quantitative research approach. The constructs in the research model are measured with scales from existing literature, with minor modifications were made to fit the current research context. The measures are reported in “Appendix A”. The constructs, except CPB and SCD, have used a 5-point Likert-scale from ‘strongly disagree’ (1), ‘neutral’ (3) to ‘strongly agree’ (5). *SCD* items use a 5-point Likert-scale of ‘not at all’ (1) to ‘to a very large extent" (5). For *CPB*, the participants report the degree to which they have seen/perceived consumers bought of each item unusually high, on a 5-point Likert-scale: 1 ‘not at all’ (1), ‘very slightly’ (2), ‘moderately’ (3), ‘to a considerable degree’ (4), and ‘very considerably’ (5).

### Data collection

Data have been collected from Australia, which have been overwhelmed with many fake news and misconceptions about COVID-19 via SM. In general, fake news and misleading information is a huge concern in Australia (Park, Fisher, McGuinness, et al., 2021b). Like worldwide (Ahmad & Murad, 2020; Al-Zaman, 2021), 23% of Australians have encountered ‘a great deal of’ (and 30% with ‘some’) misinformation about COVID-19, and for 66% of people such misinformation are obtained from SM, mostly Facebook (Park, Fisher, Lee, et al., 2021a; Park, Fisher, McGuinness, et al., 2021b). “Facebook has removed more than 110,000 pieces of COVID-related misinformation generated by Australian accounts in the first year of the pandemic” (Taylor, 2021). Also, the cases of CPB in all over Australia have been repetitive (Chau et al., 2021), affecting the retail SC regularly (Ho, 2020). The respondent profile is presented in Table 1.

**Table 1:** The respondent profile (after screening; n = 185)

Property	%	Property	%
**Gender**			
Male	57		
Female	43		
**Age**		**Facebook use**	
18–24	13	Once a week	2
25–34	23	2–4 times a week	7
35–44	26	5–6 times a week	11
45–54	22	Once a day	31
55–64	12	2–3 times a day	28
65 and above	4	4–5 times a day	15
		> than 5 times a day	6
**Education**		**Role in SC**	
Less than high school	3	Retailer	32
High school	34	Manufacturer	14
College degree	28	Supplier	35
Bachelor’s degree	15	Distributors	19
Master’s degree	6		
Doctoral degree	0		
Professional degree	14		

An online survey was used to collect data. As some of the constructs (e.g. social media fake news, panic buying behaviour) in our model have scale items with self-reporting type statements (i.e., respondents report about their actions) (Fernandez-Ballesteros, 2003), self-reporting questionnaire was included in the survey instrument. Data were collected from the retail SC network members in Australia (e.g., food and grocery retailers/supermarkets, their suppliers, manufacturers, and distributors). Online questionnaire survey was administered among the retail SC members with the help of relevant professional associations and groups in Australia such as Grocery Products Manufacturers Association, Food Distributors Association, Retailers Association, and Retailer Meet-up Groups. An invitation to participate the survey, with a survey link, was sent to 442 potential respondents. At the beginning of the survey, respondents were asked to answer screening questions to determine their eligibility to participate. In particular, they were asked to indicate if they had an active account on Facebook; next, if they had visited the account during the past three months. We filtered out respondents who did not pass these screening questions. Following the screening questions, respondents were asked to complete the questionnaire that included demography and measures of the variables. Total 191 responses were received from the survey and out of them 185 usable responses were found after screening out responses with significant missing values.

### Common method bias test

Before proceeding to data analysis, We applied procedural remedies to minimize and statistical tests to check CMB (Podsakoff et al., 2003). Regarding procedural remedies, we used already developed and tested measurement items. Yet, we conducted a pre-test with three academicians, two PhD students and two SC practitioners to refine the items that found unclear. In the survey, we kept the instructions simple, specific, and concise. Also, respondents were not allowed to revisit the responses they already made (Chan et al., 2019). Regarding statistical remedies, first, the Harman’s one-factor test showed that the first construct only accounted for 30.58% of the variance. Second, applying marker variable (MV) technique, a MV (colour choice: “I like blue cloths, I prefer blue to other colours”), which is theoretically unrelated to the nomological network, was included in the model. The result showed an insignificant effect of the MV on CPB (*β* = 0.079, *p* > 0.05). Third, in the correlation matrix, the correlation between MV and other variables was significantly below the 0.9 threshold. Taken together, the tests provide evidence for the minimal threat of CMB in our data. To minimize the threat of social desirability bias, respondents were assured in the consent form that their responses would be anonymous and kept confidential.

## Data analysis and findings

To operationalize the research objectives and to test the hypotheses in the research model, we use component-based SEM that is PLS-SEM. PLS modelling is based on an algorithm that firstly estimates the best weights of each block of the measurement model, and then the path coefficients in the structural model (Chin & Newsted, 1999). Thus, the latent variable component scores depend on how well the measurement model and structural model are specified. PLS also can handle complex model analysis (e.g., with multiple moderators) with small sample sizes under non-normality conditions (Hair Jr et al., 2021), which suits the sample size and constructs of this study.

### Assessing the measurement model

Following standard PLS procedure, the validity of the constructs was established by examining their reliability, convergent validity, and discriminant validity. For internal consistency, composite reliability (CR) for each construct was calculated. As shown in Table 2, all the values for CR are greater than the threshold of 0.70. In order to evaluate convergent validity of the constructs, outer loadings of the items and the average variance extracted (AVE) were checked (Hair Jr et al., 2017). Following Igbaria et al. (1995), three items having loading below 0.6 were discarded (see “Appendix A”). The model was run again and found that all constructs achieved the acceptable value of AVE of >  = 0.5 (see Table 2). Following Bentall et al. (2021), the measurement of CPB was treated as single item (as a proxy to the average score of all items where they measured CPB as the extent to which consumers bought the essential goods unusually high). Given a single item measurement of CPB, its item loading, CR, and AVE are not shown. Discriminant validity was assessed based on correlations and square root of AVE score of the constructs (Fornell and Larcker 1981) (see Table 2). The collective evidence suggests that the constructs and the items demonstrate good measurement properties.

**Table 2:** Psychometric properties of the constructs

	Construct correlations for discriminant validity test													
	CR	AVE	ISA	CTA	EDA	ADA	FN	BLV	SCD	FLX	RNC	VSB	CLB	CPB
ISA	0.922	0.747	0.864											
CTA	0.941	0.842	0.735*	0.918										
EDA	0.890	0.731	0.758*	0.720*	0.855									
ADA	0.833	0.626	0.735*	0.693*	0.697*	0.791								
FN	0.889	0.728	0.851*	0.576*	0.657*	0.593*	0.853							
BLV	0.819	0.608	− 0.065	0.006	− 0.004	− 0.072	0.020	0.780						
SCD	0.898	0.687	0.694*	0.439*	0.519*	0.476*	0.813*	0.050	0.829					
FLX	0.864	0.517	0.571*	0.633*	0.584*	0.664*	0.436*	− 0.049	0.322*	0.719				
RNC	0.889	0.729	0.720*	0.463*	0.455*	0.485*	0.736*	− 0.067	0.627*	0.342*	0.854			
VSB	0.811	0.588	0.614*	0.604*	0.519*	0.575*	0.471*	− 0.027	0.294*	0.583*	0.339*	0.767		
CLB	0.802	0.574	0.631*	0.633*	0.637*	0.617*	0.508*	− 0.068	0.410*	0.563*	0.388*	0.630*	0.758	
CPB	–	–	0.530*	0.429*	0.415*	0.398*	0.581*	0.064	0.624*	0.298*	0.443*	0.304*	0.347*	1

ISA, information-sharing affordance; CTA, connectivity affordance; EDA, editability affordance; ADA, association-denial affordance; FN, fake news; BLV, Believability; SCD, supply chain disruption; FLX, flexibility; RNC, redundancy capability; VSB, visibility; CLB, collaboration; CPB, consumer panic behaviour

Significance at *p < 0.001

### Assessing the structural model

As the assessment of the structural model, applying standard PLS bootstrapping method, we calculated the explained variance (i.e., *R*^*2*^) values of the endogenous constructs; and path coefficients (*β)*, *t*-statistics and *p* values. To test moderation effects, we followed two-stage approach using SmartPLS because it “is versatile and should generally be given preference for creating the interaction term” (Hair Jr et al., 2017, p. 263). Before we ran the moderation analysis, we first checked the measurement properties of the moderators. According to Appendix 1 and Table 2, all values of the moderators (item loading, CR, and AVE) are above the threshold limit. Further, Table 2 indicates that the inter-correlations among the moderators and the other variables are satisfactory. In the two-stage approach, we used the ‘moderating effect’ function in SmartPLS and chose the ‘standardised’ product term generation method and ‘automatic’ weighing mode. The significance of a moderator was evaluated with the *β, t, and p* values (see Table 3).

**Table 3:** Significance testing results of the structural model

Relationship	*β* value	*t* value	*p* value	Result
ISA to FN	0.727	8.749***	0.000	Accepted
CTA to FN	0.140	2.409*	0.016	Accepted
EDA to FN	0.270	3.010**	0.003	Accepted
ADA to FN	0.111	2.314*	0.021	Accepted
FN to CPB	0.580	13.285***	0.000	Accepted
CPB to SCD	0.394	6.265***	0.000	Accepted
FN x BLV on CPB	− 0.054	0.719	0.472	Rejected
CPB x FLX on SCD	− 0.080	1.415	0.158	Rejected
CPB x VSB on SCD	− 0.219	1.264	0.175	Rejected
CPB x CLB on SCD	− 0.010	1.082	0.942	Rejected
CPB x RNC on SCD	− 0.021	0.439	0.661	Rejected

ISA, information-sharing affordance; CTA, connectivity affordance; EDA, editability affordance; ADA, association-denial affordance; FN, fake news; BLV, Believability; CPB, consumer panic buying; FLX, flexibility; VSB, visibility; CLB, collaboration; RNC, redundancy capability

Significance at *p < 0.05, **p < 0.01, ***p < 0.001

**Fig. 2:**
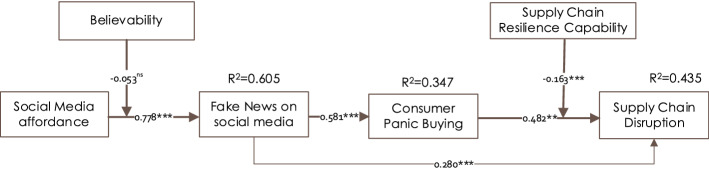
Results of the structural model considering affordance and resilience capability as higher-order constructs. Significance at *p < 0.05, **p < 0.01, ***p < 0.001

As a comprehensive analysis, first, we examined the assessment properties of the higher-order model (considering *affordance* and *SCR capability* as higher-order constructs, each having their four lower-order constructs), shown in Fig. 2. The results suggest that the *R*^*2*^ value for SM fake news, CPB, and SCD are substantial, moderate, and weak (Henseler et al., 2009). Although the *R*^*2*^ value for SCD is low, it is common in behavioural research (Minitab, 2013). Nonetheless, it exceeds the 0.10 threshold (Falk & Miller, 1992) and is significantly different from 0, indicating that our model has statistically significant explanatory power (Paetzold, 2016).

Except the moderating effect of believability, all other relations are supported. The results (see Fig. 2) suggest that SM affordances positively influence SM fake news, which leads to CPB; in turn, higher CPB results higher SCD*.* In addition, the moderation test reveals that, the effect of CPB on SCD is decreased with SCR capabilities.

**Fig. 3:**
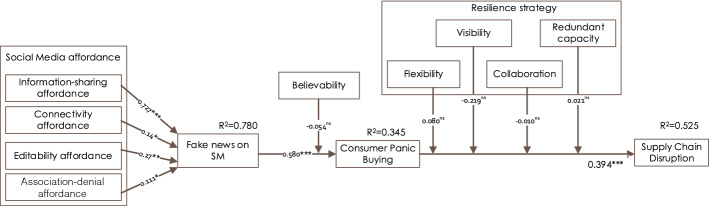
The structural results of the full model. Significance at *p < 0.05, **p < 0.01, ***p < 0.001

Next, we examined the model considering *affordance* and *SCR capabilities* as reflective-formative second-order constructs by applying PLS path modelling through repeated use of the items of the lower-order constructs (Hair Jr et al., 2017). For example, the total 13 items of ISA, CTA, EDA, and ADA have been used to measure *SM affordances*. Figure 3 and Table 3 summarise the hypotheses test results. The results suggest that the four affordances drive to fake news on SM, which leads to CPB; in turn, CPB leads to SCD. However, believability does not moderate the relationship between SM affordance and SM fake news; and none of the SCR capabilities *individually* moderates the effect of CPB on SCD.

## Post hoc analyses

### Configurational effects of resilience capabilities

It is interesting that SCR capability, as a higher-order construct, had a moderating effect between CPB and SCD (Fig. 2); however, the individual capabilities did not show any significant moderating effect (Fig. 3). To find out more, we checked their configurational effects using fuzzy set qualitative comparative analysis (fsQCA) (Ragin, 2008). FsQCA has been recently applied in various studies related to social media (Hossain et al., 2022; Pappas et al., 2020), however it is less often applied as a post hoc analysis. For a tutorial and a detailed step-by-step procedure we refer the reader to the work of Pappas and Woodside (2021). Here we follow the same approach and the recommended guidelines.

First, using fsQCA software, we perform *data calibration*. As fsQCA relies on Boolean algebra (presence/absence of constructs), Likert scale data needed to be calibrated from a crisp value (1 to 5) into a fuzzy form (between 0 and 1). As we used a five-point Likert scale, 4, 3, and 2 are used for full membership, crossover, and full non-membership (Pappas & Woodside, 2021). Second, we checked the ‘necessary condition’. A condition is considered as ‘necessary’ if its associated consistency is >  = 0.9. Also, if such conditions exist it is important to examine the Relevance of Necessity (RoN) (Schneider & Wagemann, 2012) Based on this, none of the capabilities was found to be the necessary for reducing SCD. This supports our prior results showing that no single SCR capability succeeds to minimise SCD. Third, we have checked the ‘sufficient conditions’ i.e., the configurations that combine the SCR capabilities to sufficiently explain the SCD. For a configuration to be considered sufficient, its consistency needs to be >  = 0.75. The fsQCA produced three configurations (C1-C3 in Table 4). According to the first configuration C1, SCD is reduced when a firm’s SC possesses high flexibility and high visibility capabilities. Similarly, C2 suggests that high SC visibility and collaboration capability together is sufficient for reducing SCD. Finally, C3 suggests that high flexibility and collaboration capacity together, even with lack of redundant capability, lowers SCD.

**Table 4:** The configurations of four resilience capabilities

Models	Raw Coverage	Unique Coverage	Consistency
C1. FLX*VSB	0.59	0.09	0.75
C2. VSB*CLB	0.55	0.08	0.75
C3. FLX*~RNC*CLB	0.28	0.13	0.76
Solution coverage	0.82		
Solution consistency	0.76		

### Assessment of the mediation effect

We further conducted bootstrapping analyses to examine the mediating effect using PLS. To begin the mediation analysis, we tested significance of the indirect effects. The indirect effect from *fake news* via CPB to SCD is the product of the path coefficients from *fake news* to CPB and from CPB to SCD. We found that the indirect effect is significant (*β* = 0.228, *t* = 5.255, *p* = 0.00). Next, we focused on the significance of the direct effect from *fake news* to SCD, which too was significant (*β* = 0.671, *t* = 13.325, *p* = 0.00). Hence, CPB partially mediates the relationship between *fake news* and SCD, which indicates that *fake news* has a direct positive effect on SCD beyond the effect that is mediated by CPB.

### Comparison with alternative models

We performed a pseudo-*F* test to assess the effects of excluding SM affordances or CPB from the model, along with the resulting change in variance explained for SCD. For this, we performed the effect size of the exogenous variables of the research model with respect to the endogenous variable (i.e., CPB, SCD*)*. It appears that the effect sizes of *f*^*2*^ range from ‘medium’ to ‘large’^2^ (*ISA* 0.338, *CTA* 0.140, *EDA* 0.210, *ADA* 0.289, *FN* 0.511, and *CPB* 0.243). We further assessed the *q*^*2*^ effect size, which was ‘large’ for all excluded variables (*ISA* 0.738, *CTA* 0.701, *EDA* 0.669, *ADA* 0.582, *FN* 0.650, and *CPB* 0.406). The effect size results show that the individual exclusion of each of these variables leads to a significant drop in variance for SCD. These results provide support the integration *moral panic theory* and *affordance theory* to explain SCD caused by SM fake news.

## Discussion

The objectives of this work are to understand to examine how SM affordances contribute to SM fake news, and to what extent SM fake news contribute to perceived SCD. We build on *moral panic theory* and the affordance perspective to develop a model that explains the role of SM affordance on SM fake news, which eventually lead to SCD. The research model has been tested using a survey with 185 Facebook users. Empirical results provide strong evidence in support of the research model. In the following sections, we discuss implications for research and practice, limitations, and avenues for future research.

### Implications for research

This work presents important implications for research. Studies have replicated and applied the *affordance theory* in a wide variety of settings. Particularly, the technology affordance perspectives have been extensively employed to study SM users’ behaviour on SM platforms (e.g., Apuke & Omar, 2021; Chan et al., 2019). Similarly, *moral panic theory* has been applied in studies to investigate people’s behaviour in crises (Cohen, 2011). However, their combined ability to illuminate peoples’ behaviour enabled by technology affordances, particularly in the context of a crisis, has yet to be investigated. This current study develops an empirically validated theoretical framework that summarises as follows. Different SM affordances lead to SM fake news, which creates consumer panic eventually to disrupt SC; however, SCR capabilities may mitigate the effect of consumer panic on SCD. The developed integrated model delineates to what extent the affordances of SM may create social panic and have a greater business effect i.e., to disrupt SC. It thus theoretically and empirically demonstrates that moral panic theory and affordance theory together is effective in explaining the current phenomenon. Such an integrated approach is useful to understand how different SM affordances may instigate fake news to generate and propagate, which eventually create social chaos and panic.

Extant studies in operations and SC management literature consider SCR as a multi-dimensional construct, formed either as a reflective (e.g., Chowdhury & Quaddus, 2017) or formative construct (e.g., Mwangola, 2018). The way that lower-order constructs form and compute a higher-order construct will influence the results and their interpretation. Variance-based methods examine such constructs in a competing environment as they compute net effects among variables in a model (Pappas & Woodside, 2021). In this paper, we take a different approach by applying fsQCA and identify how the different SCR capabilities and their combinations in a synergetic manner can be necessary or sufficient to explain the outcome. In other words, this study offers a configurational approach to SCR studies and suggests that combinations of the resilience, rather than an individual resilience, explain the effect of SCR on SCD better. For example, success (e.g., reduction in SCD) can be obtained by different combinations of SCR capabilities (e.g., flexibility, and visibility; or visibility and collaboration), which do not include all the SCR capabilities. Instead, our findings show that by taking a configurational approach, we can identify which combinations of SCR capabilities can equally explain the same outcome. Our study thus advances the knowledge of SCR by using a configurational lens suggesting equifinal means towards mitigating SCD. Employing fsQCA can help explain inconsistent effects of SC resilience capabilities identified by prior studies (Iftikhar et al., 2021) that focus only on net effects among variables, thus complementing and extending relevant studies in the area. It offers deeper insight into how SCR capabilities should combine within to reduce SCD, which aids researchers to revisit their models and check if a configurational model can better explain their research problem and the context (e.g., Chowdhury & Quaddus, 2017).

### Implications for practice

Our study explores the effects of SM fake news on consumers’ panic buying and consequent effect on SC. The results provide insightful practical implications, specifically for SC players and SM administrators.

Our research offers implications to SC operations. Despite the relatively large attention from industry and academia about the nasty consequence of CPB, few studies have examined companies’ resilience capabilities when there is disruptions in SC. Specifically, “The inability of SCs to cope with the situation during the pandemic has explicitly put forward a lack of expertise and research to mitigate the risk of panic buying during emergency situations or disasters” (Dulam et al., 2021, p. 4370). Therefore, this research can be an important foundation for organisational preparedness to strengthen the efficiency of SC performance during crises.

The empirical findings of this work provide insights into how to minimize the effect of CPB on SCD with different resilience capabilities. Specifically, our findings suggest that flexibility and redundancy capabilities are highly instrumental in streamlining SC operations and being resilient against the deleterious effect of SCD due to consumer panic. Hence, SC managers should strengthen flexibility and redundant capabilities to manage disruption. In addition, visibility and collaboration capabilities may not be helpful to mitigate disruptions without flexibility and redundant capabilities. This research will assist the operations and SC managers to strategize and understand which combination of resilience capabilities is the most effective one in tackling disruption during a large-scale crisis. Academicians and researchers too can use our approach to understand the configurational effect of formative variables e.g., dynamic capabilities to enhance firm performance (e.g., Wilden et al., 2013).

This study also has implications for managers controlling SM fake news. While many countries across the globe are increasingly aware of the threats posed by SM fake news but are struggling to find effective ways to reduce it and its spread (Robinson et al., 2019). In a similar vein, although previous studies have enumerated the serious consequences of SM fake news, before this current study, we did not know much about its enablers. As antibiotics are made from the virus itself, the problem of tech-based misinformation has to be solved through technology (Kreps, 2020). The SM affordances from our study provide insights into how to combat fake news SM. Such knowledge can guide the SM guardians to identify techniques to mitigate fake news on SM.

We found *association-denial* affordance is the strongest determinant of SM fake news. Hence, SM platforms need to develop mechanisms to efficiently identify the users who are involved on generating and spreading fake news and thus limit the spread of disinformation (Robinson et al., 2019). Technologies such as artificial intelligence (AI) and data-mining algorithms can be used to discover the source of fake news, against which the administrators of specific platform can take operational (e.g., suspend and delete account) and legal actions for public deceit to deter future potential offenders (Ahinkorah et al., 2020). Similarly, SM administrators can use digital technologies including AI, machine learning etc., (Kreps, 2020) to review posts and trace back a source of fake news, going beyond the editability affordances.

Our research suggests that *information-provision* is the second-most important affordance; ‘The best way to fight misinformation is to swamp the landscape with accurate information … answer people’s questions and, ultimately, fears.” (Wardle, 2020). In this current age, SM play a pivotal role in (re)forming and (re)modelling peoples’ thoughts and behaviour by providing correct news (and thus dispel fake ones). This is even more critical during situations of crises including COVID-19 (Loxton et al., 2020). Therefore, myth busting and authentic news and COVID-19-related (market) information from various regimented bodies should be provided on SM platforms. For instance, in Australia, anyone can access Facebook ‘live list’ to follow the facts and discussion on COVID-19, which potentially reduce pubic confusion and fake news (Taylor, 2021).

### Limitations and future research directions

This study provides an integrated approach to understand how different SM affordances may instigate fake news, leading to social chaos and panic. This calls for research into other circumstances and contexts including the effect of SM misinformation on anti-vaccination campaign, brand hate and avoidance, and racial and religious unrest and/or riots. We further expect that the limitations our study will stimulate future research.

First, we have dealt with perceived SM fake news on COVID-19 than the actual fake news. Future studies can either observe phenomena or run experiments to explore how and why an actual SM content (e.g., fake news) spreads, and how then it affects the existent businesses (e.g., spikes in supply/demand). Given that, moral panic is short-lived (which we see that people’s (re)action on COVID-19 and its associated matters change over time), a longitudinal study incorporated in different periods during the pandemic will be interesting. Furthermore, collecting posts from social media and employing natural language processing (NLP) can offer a different perspective towards the creation of more effective pandemic management strategies (Choudrie et al., 2021; Kang et al., 2020). Our work lays a foundation on such future research.

Our second limitation is based on the theory of bounded rationality (Simon, 2000). We assume that, while responding to the measures of SCR capabilities, the respondents i.e., SC professionals have prioritised the capabilities which are not necessarily perfectly rational, constrained by the intractability of decision process and finite information available to them. Hence, the combinations of the resilience capabilities (i.e., configurations) are ‘satisfactory’ and ‘sufficient’ than ‘optimal’ or ‘necessary’. Therefore, SC managers should apply the combinations of the SCR capabilities cautiously and future research can further investigate if our configurations are consistent over time, space, and contexts.

Third, we have collected data from a developed country i.e., Australia. Although panic-buying is believed to take place mostly in developed countries (Sim et al., 2020) where people are with ‘comfortable circumstances’ and thus averse to deal with uncertainties (Bentall et al., 2021), it can happen in developing countries as well (Singh et al., 2021). The nature and impact of panic buying can even vary between the people living in urban areas compared to those living in rural areas (Bentall et al., 2021), and between wealthier households and their impoverished counterparts (O'Connell et al., 2021). Future studies can explore if the variation of economic outlook of a country and among consumers has any role on panic behaviour and associated factors.

Fourth, while the *affordance theory* has been useful for our purpose i.e., to identify the factors that influence the prevalence of SM fake news, it is only one approach for identifying the enablers. Other theoretical approaches could be valuable for identifying additional drivers. Similarly, *moral panic theory* has provided us the theoretical underpinning to understand people’s behaviour in exaggerated public problem, done by the means of media, whereas other theoretical approaches can be used as well. For example, *chaos theory* (e.g., Abraham & Gilgen, 1995) could explain people’s behaviour during the early stage of COVID-19 where chaos was existent everywhere e.g., health, education, and social systems. Similarly, to explain how contagion may influence the circulation of fake news on SM platforms (Chua et al., 2021), *contagion theory* (Locher, 2002) or *deindividuation theory* (Diener, 1977) would be a reference theory.

Fifth, our list of SM affordances and SCR capabilities, and the source and effect of CPB are not exhaustive. For instance, SCD during COVID-19 can occur for various other reasons than CPB including restricted production and transportation because of lock down (Katsaliaki et al., 2021). Similarly, CPB can have varied effects e.g., on production and freight capacity dynamics (Chua et al., 2021). Future research can build on our model and extend. Finally, the issues examined in this article should also be examined in relation to emerging technologies including artificial intelligence and metaverse (Dwivedi et al., 2021a, 2021b; 2022). For example, future studies should explore “*how does the misinformation, disinformation, and fake news share and spread in the metaverse?*” (Dwivedi et al., 2022, p. 48) and how different affordances of the metaverse can contribute to fake news.

## Conclusion

This study investigates how SM fake news leads to SCD during COVID-19, and how different SM affordances contribute to fake news. Our findings indicate that, SM affordances positively contribute to SM fake news, which increases CPB. Eventually, CPB leads to SCD. Yet, the impact of CPB on SCD can be mitigated by employing specific combination of resilience capabilities. This study offers practical implications. For instance, it suggests that fake news on SM is possible because of four SM affordances; by controlling them properly, we can reduce fake news. As a mechanism to reduce the effect of fake news on SCD, it suggests different combinations of SCR capabilities. In the theoretical realm, this study extends existing knowledge by integrating *affordance theory* and *moral panic theory* to explain SM affordances, and CPB and its consequence, respectively and thus provides an insightful theoretical lens for future research. However, further investigation is needed on how the consumers in developing countries react to SM fake news and whether they participate in panic buying. Moreover, the SCR capabilities may need to customise in those countries to reduce the effects of fake news on SCD.

## Appendix A: The measures

**Table:**

Construct	Items	Loading
Information-provision affordance	Online social media offer me the possibility to…	
	ISA1: share valuable content	0.875
	ISA2: share useful content	0.909
	ISA3: share knowledge	0.845
	ISA4: disseminate information	0.826
Connectivity affordance	Online social media offer me the possibility to…	
	CTA1: reach a social media user	0.903
	CTA 2: to get in touch with a social media user	0.940
	CTA 3: to connect with a social media user	0.910
Editability affordance	Online social media offer me the possibility to…	
	EDA1: modify the contents I have posted	0.818
	EDA2: revise the materials I have uploaded	0.812
	EDA3: amend the information I have created	0.910
Association denial affordance	Online social media offer me the possibility to…	
	ADA1: associate the agency of my posts to other users who have interacted with them	0.900
	ADA2: shift the ownership of my contents among other users who have interacted with them	0.755
	ADA3: attribute the accountability of my materials shared on the platform to other users	0.878
Fake news on SM	On online social media, I saw:	
	FS1. information related to COVID-19 that I later found out as a hoax	0.852
	FS2. content related to COVID-19 that seem accurate at a time and I later found was made up	0.894
	FS3. content related to COVID-19 that was exaggerated	0.811
Believability	On social media, I saw contents related to COVID- 19 that are:	
	BLV1. believable	0.665
	BLV2. truthful	0.964
	BLV3. credible	0.674
Consumer Panic Buying	Please indicate the degree to which you have seen/perceived customers increased their purchasing (i.e., bought more than usually) of the following items during the COVID-19 pandemic: Tinned foods Water Sanitary products (e.g., hand sanitiser) Toilet roll and/or hand towels Dried food (e.g., pasta, rice) Bread Pharmacy products (e.g., painkillers, cold/flu products) Batteries Fuel (heating or car fuel)	-
Supply Chain Disruption	How did the disruption due to “panic buying in COVID-19" negatively affect (directly or indirectly) your business unit and its SC on the following dimensions?	
	SCD1. Overall efficiency of our operations	0.820
	SCD2. Procurement costs/Prices for the purchased item	0.866
	SCD3. Product quality of our final product(s)	0.842
	SCD4. Production quantity*	…
	SCD5. Responsiveness to customer demands. SCD6. Delivery reliability (on-time delivery, order accuracy)	0.784
Supply Chain Flexibility Capacity	FLX1. We have flexibility in production	0.775
	FLX2. We produce different types of products to meet customer requirements	0.789
	FLX3. We have multi-skilled workforce to continue our operations*	…
	FLX4. We have contract flexibility such as partial order, partial payment, partial shipment etc	0.714
	FLX5. We have flexibility in sourcing	0.736
	FLX6. We have flexibility in distribution	0.692
	FLX7. We are capable of introducing new product	0.601
Supply Chain Visibility Capacity	VSB1. We share information with SC partners	0.801
	VSB2. We track information of operations in the SC	0.757
	VSB3. We have business intelligence systems	0.741
	VSB4. We have real-time flow of information with SC members*	…
Supply Chain Redundant Capacity	RNC1. We have back up capacity	0.867
	RNC2. We have buffer stock	0.912
	RNC3. We have backup energy/utility source*	0.776
Supply Chain Collaboration Capacity	CLB1. We have collaborative forecasting of demand with SC partners	0.752
	CLB2. We have collaborative planning & decision making practice with the SC partners	0.756
	CLB3. We invest in our suppliers’ plant to collaborate operations	0.766

“*” represents discarded item because of low loading

## Footnotes

1 SM affordances are not generalised but may vary with a medium e.g., Facebook and Tiktok. As this current study conducts survey among Facebook users, the affordances are more applicable to this medium.

2 **Note**: *f*^*2*^ ≥ 0.02*, f*^*2*^ ≥ 0.15*,* and *f*^*2*^ ≥ 0.35 represent weak, medium, and large effect sizes, respectively Henseler, J., Ringle, C. M., & Sinkovics, R. R. (2009). The use of partial least squares path modeling in international marketing. In *New challenges to international marketing*. Emerald Group Publishing Limited.
